# pysradb: A Python package to query next-generation sequencing metadata and data from NCBI Sequence Read Archive

**DOI:** 10.12688/f1000research.18676.1

**Published:** 2019-04-23

**Authors:** Saket Choudhary

**Affiliations:** 1 Computational Biology and Bioinformatics, University of Southern California, Los Angeles, California, 90089, USA

**Keywords:** bioinformatics, metadata, SRA, NGS, NCBI, GEO

## Abstract

The NCBI Sequence Read Archive (SRA) is the primary archive of next-generation sequencing datasets. SRA makes metadata and raw sequencing data available to the research community to encourage reproducibility and to provide avenues for testing novel hypotheses on publicly available data. However, methods to programmatically access this data are limited. We introduce the Python package, pysradb, which provides a collection of command line methods to query and download metadata and data from SRA, utilizing the curated metadata database available through the SRAdb project. We demonstrate the utility of pysradb on multiple use cases for searching and downloading SRA datasets. It is available freely at https://github.com/saketkc/pysradb.

## Introduction

Several projects have made efforts to analyze and publish summaries of DNA- ^1^ and RNA-seq ^2, 3^ datasets. Obtaining metadata and raw data from the NCBI Sequence Read Archive (SRA) ^4^ is often the first step towards re-analyzing public next-generation sequencing datasets in order to compare them to private data or test a novel hypothesis. The NCBI SRA toolkit ^5^ provides utility methods to download raw sequencing data, while the metadata can be obtained by querying the website or through the Entrez efetch command line utility ^6^. Most workflows analyzing public data rely on first searching for relevant keywords in the metadata either through the command line utility or the website, gathering relevant sample(s) of interest and then downloading these. A more streamlined workflow can enable the performance of all these steps at once.

In order to make querying both metadata and data more precise and robust, the SRAdb ^7^ project provides a frequently updated SQLite database containing all the metadata parsed from SRA. SRAdb tracks the five main data objects in SRA’s metadata: submission, study, sample, experiment and run. These are mapped to five different relational database tables that are made available in the SQLite file. The metadata semantics in the file remain as they are on SRA. The accompanying package, SRAdb ^8^, made available in the R programming language ^9^, provides a convenient framework to handle metadata queries and raw data downloads by utilizing the SQLite database. Though powerful, SRAdb requires the end user to be familiar with the R programming language and does not provide a command-line interface for querying or downloading operations.

The pysradb package ^10^ builds upon the principles of SRAdb, providing a simple and user-friendly commandline interface for querying metadata and downloading datasets from SRA. It obviates the need for the user to be familiar with any programming language for querying and downloading datasets from SRA. Additionally, it provides utility functions that will further help a user perform more granular queries, which are often required when dealing with multiple datasets on a large scale. By enabling both metadata search and download operations at the command-line, pysradb aims to bridge the gap in seamlessly retrieving public sequencing datasets and the associated metadata.

pysradb ^10^ is written in Python ^11^ and is currently developed on GitHub under the open-source BSD 3-Clause License. To simplify the installation procedure for the end-user, it is also available for download through PyPI and bioconda ^12^.

## Methods

### Implementation

pysradb ^10^ is implemented in Python and uses pandas ^13^ for data frame based operations. Since downloading datasets can often take a long time, pysradb displays progress for long haul tasks using tqdm ^14^. The metadata information is read in the form of an SQLite ^15^ database, made available by SRAdb ^7^.

Each sub-command of pysradb contains a self-contained help string that describes its purpose and usage example. The help text can be accessed by passing the ‘–help’ flag. There is also additional documentation available for the sub-commands on the project’s website. We also provide example Jupyter ^16^ notebooks that demonstrate the functionality of the Python API.

pysradb’s development primarily occurred on GitHub and the code is tested continuously using Travis CI webhook. This monitors all incoming pull requests and commits to the master branch. The testing happens on Python version 3.5, 3.6, and 3.7 on an Ubuntu 16.04 LTS virtual machine, while testing webhooks on the bioconda channel provide additional testing on Mac-based systems. Nevertheless, pysradb should run on most Unix derivatives.

### Operation

pysradb ^10^ can be run on either Linux- or Mac-based operating systems. It supports Python 3.5, 3.6 and 3.7. Requiring just two additional dependencies, pysradb can be easily installed using either a pip- or conda-based package manager via the bioconda ^12^ channel.

An earlier version of this article can be found on bioRxiv https://doi.org/10.1101/578500

## Use cases

pysradb ^10^ provides a chain of sub-commands for retrieving metadata, converting one accession to other and downloading. Each sub-command is designed to perform a single operation by default, while additional operations can be performed by passing additional flags. In the following section we demonstrate some of the use cases of these sub-commands.

pysradb uses SRAmetadb.sqlite, a SQLite file produced and made available by SRAdb ^7^ project. The file itself can be downloaded using pysradb as:



                    $ pysradb srametadb
                


The SRAmetadb.sqlite file is required for all other operations supported by pysradb. This file is required for all the sub-commands to function. By default, pysradb assumes that the file is located in the current working directory. Alternatively, it can supplied using the ‘–db path/to/SRAmetadb.sqlite’ argument. The SRAmetadb.sqlite is available at: https://s3.amazonaws.com/starbuck1/sradb/SRAmetadb.sqlite.gz or alternatively at https://gbnci-abcc.ncifcrf.gov/backup/SRAmetadb.sqlite.gz. The examples here were run using SRAmetadb.sqlite with schema version 1.0 and creation timestamp 2019-01-25 00:38:19.

### Search

Consider a case where a user is looking for Ribo-seq ^17^ public datasets on SRA. These datasets will often have ‘ribosome profiling’ appearing in the abstract or sample description. We can search for such projects using the ‘search’ sub-command:



                        $ pysradb search ‘"ribosome profiling"’ | head
                    


**Table:** 

study_accession	experiment_accession	sample_accession	run_accession
DRP003075	DRX019536	DRS026974	DRR021383
DRP003075	DRX019537	DRS026982	DRR021384
DRP003075	DRX019538	DRS026979	DRR021385
DRP003075	DRX019540	DRS026984	DRR021387
DRP003075	DRX019541	DRS026978	DRR021388
DRP003075	DRX019543	DRS026980	DRR021390
DRP003075	DRX019544	DRS026981	DRR021391
ERP013565	ERX1264364	ERS1016056	ERR1190989

The results here list all relevant ‘ribosome profiling’ projects.

### Getting metadata for a SRA project

Each SRA project (accession prefix ‘SRP’) on SRA consists of single or multiple experiments (accession prefix ‘SRX’) which are sequenced as single or multiple runs (accession prefix ‘SRR’). Each experiment is carried out on an individual biological sample (accession prefix ‘SRS’).

pysradb metadata can be used to obtain all the experiment, sample, and run accessions associated with a SRA project as:



                        $ pysradb metadata SRP010679 | head
                    


**Table:** 

study_accession	experiment_accession	sample_accession	run_accession
SRP010679	SRX118285	SRS290854	SRR403882
SRP010679	SRX118286	SRS290855	SRR403883
SRP010679	SRX118287	SRS290856	SRR403884
SRP010679	SRX118288	SRS290857	SRR403885
SRP010679	SRX118289	SRS290858	SRR403886
SRP010679	SRX118290	SRS290859	SRR403887
SRP010679	SRX118291	SRS290860	SRR403888
SRP010679	SRX118292	SRS290861	SRR403889
SRP010679	SRX118293	SRS290862	SRR403890
SRP010679	SRX118294	SRS290863	SRR403891
SRP010679	SRX118295	SRS290864	SRR403892
SRP010679	SRX118296	SRS290865	SRR403893

However, this information by itself is often incomplete. We require detailed metadata associated with each sample to perform any downstream analysis. For example, the assays used for different samples and the corresponding treatment conditions. This can be done by supplying the ‘–desc’ flag:



                        $ pysradb metadata SRP010679 –desc | head -5
                    


**Table:** 

study_accession	experiment_accession	sample_accession	run_accession	sample_attribute
SRP010679	SRX118285	SRS290854	SRR403882	source_name: PC3 human prostate cancer cells || cell line: PC3 || sample type: polyA RNA || treatment: vehicle
SRP010679	SRX118286	SRS290855	SRR403883	source_name: PC3 human prostate cancer cells || cell line: PC3 || sample type: ribosome protected RNA || treatment: vehicle
SRP010679	SRX118287	SRS290856	SRR403884	source_name: PC3 human prostate cancer cells || cell line: PC3 || sample type: polyA RNA || treatment: rapamycin
SRP010679	SRX118288	SRS290857	SRR403885	source_name: PC3 human prostate cancer cells || cell line: PC3 || sample type: ribosome protected RNA || treatment: rapamycin

This can be further expanded to reveal the data in ‘sample_attribute’ column into separate columns via ‘–expand’ flag. This is most useful for samples that have associated treatment or cell type metadata available.



                        $ pysradb metadata SRP010679 –desc –expand
                    


**Table:** 

... [truncated]				
run_accession	cell_line	sample_type	source_name	treatment
SRR403882	pc3	polya rna	pc3 human prostate cancer cells	vehicle
SRR403883	pc3	ribosome protected rna	pc3 human prostate cancer cells	vehicle
SRR403884	pc3	polya rna	pc3 human prostate cancer cells	rapamycin
SRR403885	pc3	ribosome protected rna	pc3 human prostate cancer cells	rapamycin
SRR403886	pc3	polya rna	pc3 human prostate cancer cells	pp242
SRR403887	pc3	ribosome protected rna	pc3 human prostate cancer cells	pp242
SRR403888	pc3	polya rna	pc3 human prostate cancer cells	vehicle
SRR403889	pc3	ribosome protected rna	pc3 human prostate cancer cells	vehicle
SRR403890	pc3	polya rna	pc3 human prostate cancer cells	rapamycin
SRR403891	pc3	ribosome protected rna	pc3 human prostate cancer cells	rapamycin
SRR403892	pc3	polya rna	pc3 human prostate cancer cells	pp242
SRR403893	pc3	ribosome protected rna	pc3 human prostate cancer cells	pp242

Any SRA project might consist of experiments involving multiple assay types. The assay associated with any project can be obtained by providing –assay flag:



                        $ pysradb metadata SRP000941 –assay | tr -s ’ ’ | cut -f5 -d ’ ’ | tail -n +2 | sort | uniq -c
                    


**Table:** 

999	Bisulfite-Seq
768	ChIP-Seq
121	OTHER
353	RNA-Seq
28	WGS

### Getting SRPs from GSE

The Gene Expression Omnibus database (GEO) ^18^ is the NCBI data repository for functional genomics data.

It accepts array and sequence-based data from gene profiling experiments. For sequence-based data, the corresponding raw files are deposited to the SRA. GEO assigns a dataset accession (accession prefix ‘GSE’) that is linked to the corresponding accession on the SRA (accession prefix ‘SRP’). It is often necessary to interpolate between the two accessions. gse-to-srp sub-command allows converting GSE to SRP:



                        $ pysradb gse-to-srp GSE24355 GSE25842
                    


**Table:** 

study_alias	study_accession
GSE24355	SRP003870
GSE25842	SRP005378

It can be further expanded to obtain the corresponding experiment and run accessions:



                        $ pysradb gse-to-srp –detailed –expand GSE100007 | head
                    


**Table:** 

study_alias	study_accession	experiment_accession	sample_accession	experiment_alias	sample_alias
GSE100007	SRP109126	SRX2916198	SRS2282390	GSM2667747	GSM2667747
GSE100007	SRP109126	SRX2916199	SRS2282391	GSM2667748	GSM2667748
GSE100007	SRP109126	SRX2916200	SRS2282392	GSM2667749	GSM2667749
GSE100007	SRP109126	SRX2916201	SRS2282393	GSM2667750	GSM2667750
GSE100007	SRP109126	SRX2916202	SRS2282394	GSM2667751	GSM2667751
GSE100007	SRP109126	SRX2916203	SRS2282395	GSM2667752	GSM2667752
GSE100007	SRP109126	SRX2916204	SRS2282396	GSM2667753	GSM2667753
GSE100007	SRP109126	SRX2916205	SRS2282397	GSM2667754	GSM2667754
GSE100007	SRP109126	SRX2916206	SRS2282400	GSM2667755	GSM2667755

### Getting a list of GEO experiments for a GEO study

Any GEO study (accession prefix ‘GSE’) will involve a collection of experiments (accession prefix ‘GSM’). We can obtain an entire list of experiments corresponding to the study using the gse-to-gsm sub-command from pysradb:



                        $ pysradb gse-to-gsm GSE41637 | head
                    


**Table:** 

study_alias	experiment_alias
GSE41637	GSM1020640_1
GSE41637	GSM1020641_1
GSE41637	GSM1020642_1
GSE41637	GSM1020643_1
GSE41637	GSM1020644_1
GSE41637	GSM1020645_1
GSE41637	GSM1020646_1
GSE41637	GSM1020647_1
GSE41637	GSM1020648_1

However, a list of GSM accessions is not useful if one is performing any downstream analysis, which essentially requires more detailed information about the metadata associated with each experiment. This relevant metadata associated with each sample can be obtained by providing gse-to-gsm additional flags:



                        $ pysradb gse-to-gsm –desc GSE41637 | head
                    


**Table:** 

study_alias	experiment_alias	sample_attribute
GSE41637	GSM1020640_1	source_name: mouse_brain || strain: DBA/2J || tissue: brain
GSE41637	GSM1020641_1	source_name: mouse_colon || strain: DBA/2J || tissue: colon
GSE41637	GSM1020642_1	source_name: mouse_heart || strain: DBA/2J || tissue: heart
GSE41637	GSM1020643_1	source_name: mouse_kidney || strain: DBA/2J || tissue: kidney
GSE41637	GSM1020644_1	source_name: mouse_liver || strain: DBA/2J || tissue: liver
GSE41637	GSM1020645_1	source_name: mouse_lung || strain: DBA/2J || tissue: lung
GSE41637	GSM1020646_1	source_name: mouse_skm || strain: DBA/2J || tissue: skeletal muscle
GSE41637	GSM1020647_1	source_name: mouse_spleen || strain: DBA/2J || tissue: spleen
GSE41637	GSM1020648_1	source_name: mouse_testes || strain: DBA/2J || tissue: testes

The metadata information can then be parsed from the sample_attribute column. To obtain more structured metadata, we can use an additional flag ‘–expand’:



                        $ pysradb gse-to-gsm –desc –expand GSE41637 | head
                    


**Table:** 

study_alias	experiment_alias	source_name	strain	tissue
GSE41637	GSM1020640_1	mouse_brain	dba/2j	brain
GSE41637	GSM1020641_1	mouse_colon	dba/2j	colon
GSE41637	GSM1020642_1	mouse_heart	dba/2j	heart
GSE41637	GSM1020643_1	mouse_kidney	dba/2j	kidney
GSE41637	GSM1020644_1	mouse_liver	dba/2j	liver
GSE41637	GSM1020645_1	mouse_lung	dba/2j	lung
GSE41637	GSM1020646_1	mouse_skm	dba/2j	skeletal muscle

### Getting SRR from GSM

gsm-to-srr allows conversion from GEO experiments (accession prefix ‘GSM’) to SRA runs (accession prefix ‘SRR’):



                        $ pysradb gsm-to-srr GSM1020640 GSM1020646
                    


**Table:** 

experiment_alias	run_accession
GSM1020640_1	SRR594393
GSM1020646_1	SRR594399

### Downloading SRA datasets

pysradb enables seemless downloads from SRA. It organizes the downloaded data following the NCBI hiererachy: ‘SRP => SRX => SRR’ of storing data. Each ‘SRP’ (project) has multiple ‘SRX’ (experiments) and each ‘SRX’ in turn has multiple ‘SRR’ (runs). Multiple projects can be downloaded at once using the download sub-command:



                        $ pysradb download -p SRP000941 -p SRP010679
                    


download also allows Unix pipes-based inputs. Consider our previous example of the project SRP000941 with different assays. However, we want to be able to download only ‘RNA-seq’ samples. We can do this by subsetting the metadata output for only ‘RNA-seq’ samples:



                        $ pysradb metadata SRP000941 –assay | grep ‘study|RNA-Seq’ | pysradb download
                    


This will only download the ‘RNA-seq’ samples from the project.

## Summary

pysradb ^10^ provides a command-line interface to query metadata and download sequencing datasets from the SRA. It enables seamless retrieval of metadata and conversion between different accessions. pysradb is written in Python 3 and is available on Linux and Mac OS. The source code is hosted on GitHub and licensed under BSD 3-clause license. It is available for installation through PyPI and bioconda.

## Data availability

### Underlying data

Dataset from DDBJ Sequence Read Archive, Accession number DRP003075: https://identifiers.org/ insdc.sra/DRP003075

Dataset from EMBL-EBI Sequence Read Archive, Accession number ERP013565: https://identifiers. org/insdc.sra/ERP013565

Dataset from Gene Expression Omnibus, Accession number GSE24355: https://identifiers.org/geo/ GSE24355

Dataset from Gene Expression Omnibus, Accession number GSE25842: https://identifiers.org/geo/ GSE25842

Dataset from Gene Expression Omnibus, Accession number GSE100007: https://identifiers.org/ geo/GSE100007 ^19^

Dataset from Gene Expression Omnibus, Accession number GSE41637: https://identifiers.org/geo/ GSE41637 ^20^

Dataset from NCBI Sequence Read Archive, Accession number SRP010679: https://identifiers.org/ insdc.sra/SRP010679 ^21^

Dataset from NCBI Sequence Read Archive, Accession number SRP000941: https://identifiers.org/ insdc.sra/SRP000941 ^22^

## Software availability

**Software available from:** https://pypi.org/project/pysradb/.

**Source code available from:** https://github.com/saketkc/pysradb.

**Archived source code at time of publication:** https://doi.org/10.5281/zenodo.2579446 ^10^.

**License:** BSD 3-Clause

## Author endorsement

Dr. Luiz O. Penalva confirms that the author has an appropriate level of expertise to conduct this research, and confirms that the submission is of an acceptable scientific standard. Dr. Luiz O. Penalva declares they have no competing interests. Affiliation: UT Health San Antonio, Children’s Cancer Research Institute, San Antonio, Texas, 78229, USA
