# Supplementary material for: pysradb: A Python package to query next-generation sequencing metadata and data from NCBI Sequence Read Archive
Source: F1000Res. 2019 Apr 23;8:532. [Version 1] doi: 10.12688/f1000research.18676.1 (PMC6505635; doi:10.12688/f1000research.18676.1)
Supplement: Peer review history

# Peer review: Reviewer response for version 1

**DOI:** 10.5256/f1000research.20450.r47516

**Published:** 2019-05-07

**Authors:** Ryan K. Dale

**Affiliations:** 1 Bioinformatics and Scientific Programming Core, National Institute of Child Health and Human Development, National Institutes of Health, Bethesda, MD, USA

﻿Pysradb is a Python package that extends the existing SRAdb R package by exposing command-line functionality such that metadata queries can be piped to other command-line tools.

 Overall this is a well-written tool with good documentation and tests. As-is, it is very useful and my comments here are only aimed at making the tool more useful.

**Downloading:**

 It would be really nice to be able to download fastqs, perhaps using SRAdb's strategy of downloading them from EBI.

 When trying to run the example from the docs, "pysradb download -p SRP000941", I got a 550 HTTP error. Upon closer inspection, some of the SRAs in this entry are no longer available, but it causes the whole download to fail. I'm not quite sure of the underlying cause—this seems to be a mismatch between what's in SRA and the metadata database even though I just downloaded a fresh database with a timestamp of a week ago.

 Two suggestions for making the download more robust. The first is to move on if there are any failures, reporting any failures at the end to stderr. The second is to provide a --list option that only prints the table with URLs such that the user can extract the URLs as needed (for example, to allocate to separate nodes on a cluster where failures can be handled by other mechanisms). With the current set of arguments, the only way to do the latter is to interactively answer "N" to the question of whether to continue downloading.

 It could be useful to just download individual SRRs; I do not see that functionality available.

**Searching:**

 An explanation of the search syntax would be helpful (e.g., SQL syntax, which allows wildcards but not regular expressions). More complex examples would be good here. Given the heterogeneity of user-entered metadata in SRA, it would be convenient to use regular expressions when searching (which appears to be possible with Python and sqlite3 using a callback function).

 I would prefer a nicer-formatted "no results found" printed to stderr rather than RuntimeWarning and UserWarning.

**Other:**

 An interface to check the timestamp from the metaInfo table of the database would be helpful.

 This may be a personal preference, but I would rather have output be tab-separated instead of requiring additional `tr -s " " | cut -d " "` commands (or awk) to do any sort of manipulation on the command line.

I have read this submission. I believe that I have an appropriate level of expertise to confirm that it is of an acceptable scientific standard.

# Peer review: Reviewer response for version 1

**DOI:** 10.5256/f1000research.20450.r47560

**Published:** 2019-04-26

**Authors:** Simon Andrews

**Affiliations:** 1 Bioinformatics Group, Babraham Institute, Cambridge, UK

Pysradb is a command line utility, written in python, which provides an easy scriptable interface for querying metadata and datasets from the SRA database.

 The authors correctly point out that interacting with GEO/SRA through the official site and APIs can be slow and frustrating, and thus having a tool which can make this process more streamlined is of great value.

 Both the paper and the documentation for the tool are well written and easy to understand.  The tool provides a series of individual operations performing queries, translations or downloads and these can be run individually or chained together through pipes (which is really nice!).

 The tool requires an initial download of the sqlite database from the SRAdb project.  Whilst I can see how this then makes all subsequent operations quick, it does mean that you have to download a >2GB file (which expands to >30GB), taking 30+ mins before you can do anything with the program.  It presumably also means that you need to re-download this file every time there is an update to the data in GEO otherwise your searches are likely to be out of date.  On our site at least, people are often getting data for papers which have just been released so this is going to entail a lot of waiting for this file to download.  It would be great if there was a way to point to a publicly accessible SQL server to do queries without having to do the local download, and then providing the option of pulling a local copy if you need greater performance.  Also having a way to do incremental updates to this file instead of re-downloading the whole thing would be nice. Neither of these is a deal breaker, but they mightn't be too hard to implement?

 The individual tools all worked as described, with the exception of the issues listed at the bottom, and the experience was generally very good with the tool.

 One frustrating limitation is that the piping support is not univeral throughout the tool.  You can pipe into the download command, but not, for example, into the metadata command.  Being able to chain operations such as:

 pysradb gse-to-srp GSE24355 | pysradb metadata | pysra download

 ..or

 pysradb search '"oocyte development"' | head | pysradb metadata

 ..would be really nice and presumably not too hard to support?

 The downloading side of the tool is very useful and probably the part which is hardest to achieve in the main SRA site.  Whilst this worked as described there are some aspects of the way it works which make it a little frustrating.  Firstly, it downloads SRA files, which hardly anyone wants - having a way to get the fastq files directly would be a really useful addition rather than having to run fastq-dump manually afterwards.  It also downloads into a structured set of folders, which makes sense, but for large downloads means your files are scattered through multiple folders which makes life harder when you want to process them.  Even the --out-dir option doesn't mean the files are in that directory, but just that it's used as a basename.  For the names of the files it would be nicer to have a name which incorporated the relevant SRR/SRX ids and maybe the user submitted sample name so that you can actually have a meaningful and complete name from the file.  For example, the types of filenames generated by SRA explorer (https://ewels.github.io/sra-explorer/) are a nice compromise between being predictable, unique and yet informative at the same time.

 If I'm being really picky I'd also quibble a bit at the choice of some of the defaults in the API.  For example, I can't see why the --desc and --expand options aren't the default for the metadata sub-program - give me everything in a nice format and let me cut that down if I don't need everything.  

 Overall this tool is really nice and will be useful for a lot of people.  With a small amount of refinement this is likely to become part of our standard toolbox.

 -----------------------

 Minor points to address:

 1) The API seems to have changed since the paper was written.  The option to download the metadata is now pysradb metadb, and not pysradb srametadb.  This is wrong in both the paper and the Jupyter notebook example on the github page and should be changed.  It might be nice to allow srametadb as a fallback if people have been using the old name?

 2) Some of the documentation is incomplete.  For example the quickstart documentation at https://saket-choudhary.me/pysradb/quickstart.html#the-full-list-of-possible-pysradb-operations doesn't list the search operation so I couldn't look up the options I had for that.

 3) The piping option is great, but on my system generates a crash if there is too much output (possibly more than the pipe buffer can hold?).  Submitted as bug #7

 4) The metadata command line in the paper is broken.  You need double dashes before the options, so --desc rather than -desc.  This seems to happen elsewhere as well and might just be an auto-format problem.

 5) If using wget to download the progress bar for downloading doesn't work.  The data comes down but the progress stays at 0%.

I have read this submission. I believe that I have an appropriate level of expertise to confirm that it is of an acceptable scientific standard.
